# Human endogenous retrovirus-enveloped baculoviral DNA vaccines against MERS-CoV and SARS-CoV2

**DOI:** 10.1038/s41541-021-00303-w

**Published:** 2021-03-19

**Authors:** Hansam Cho, Yuyeon Jang, Ki-Hoon Park, Hanul Choi, Aleksandra Nowakowska, Hee-Jung Lee, Minjee Kim, Min-Hee Kang, Jin-Hoi Kim, Ha Youn Shin, Yu-Kyoung Oh, Young Bong Kim

**Affiliations:** 1 KR BioTech, Seoul, Republic of Korea; 2 Department of Bio-industrial Technologies, Konkuk University, Seoul, Republic of Korea; 3 Department of Biomedical Science and Engineering, Konkuk University, Seoul, Republic of Korea; 4 Department of Stem Cell and Regenerative Biotechnology, Konkuk University, Seoul, Republic of Korea; 5 College of Pharmacy and Research Institute of Pharmaceutical Sciences, Seoul National University, Seoul, Republic of Korea

**Keywords:** DNA vaccines, SARS-CoV-2

## Abstract

Here we report a recombinant baculoviral vector-based DNA vaccine system against Middle East respiratory syndrome coronavirus (MERS-CoV) and the severe acute respiratory syndrome coronavirus-2 (SARS-CoV2). A non-replicating recombinant baculovirus expressing the human endogenous retrovirus envelope gene (AcHERV) was constructed as a DNA vaccine vector for gene delivery into human cells. For MERS-CoV vaccine construction, DNA encoding MERS-CoV S-full, S1 subunit, or receptor-binding domain (RBD) was inserted into the genome of AcHERV. For COVID19 vaccine construction, DNA encoding SARS-CoV2 S-full or S1 or a MERS-CoV NTD domain-fused SARS-CoV2 RBD was inserted into the genome of AcHERV. AcHERV-DNA vaccines induce high humoral and cell-mediated immunity in animal models. In challenge tests, twice immunized AcHERV-MERS-S1 and AcHERV-COVID19-S showed complete protection against MERS-CoV and SARS-CoV2, respectively. Unlike AcHERV-MERS vaccines, AcHERV-COVID19-S provided the greatest protection against SARS-CoV2 challenge. These results support the feasibility of AcHERV-MERS or AcHERV-COVID19 vaccines in preventing pandemic spreads of viral infections.

## Introduction

Severe acute respiratory syndrome coronavirus-2 (SARS-CoV2), the virus responsible for the unprecedented COVID19 (coronavirus disease 2019) pandemic, has prompted an urgent global effort to develop effective vaccines. Currently, at least 321 vaccine candidates are in preclinical or clinical development^1–3^. Middle East Respiratory Syndrome Coronavirus (MERS-CoV), which ultimately spread to 27 countries, is the cause of a lethal (~35% mortality rate) acute respiratory infection in humans^4^. Both SARS-CoV2 and MERS-CoV are zoonotic viral pathogens belonging to the family *Coronaviridae*, genus ß-coronavirus. The S protein of coronaviruses is known to play a critical role in inducing neutralizing antibodies and antiviral T-cell responses^5,6^.

Various viral vectors have been studied for delivery of DNA encoding S protein antigen^7–22^. Because they target human cellular receptors and are capable of translocating the target gene from the cytoplasm to the nucleus, DNA viral vector vaccines provide the most efficient gene transfer function.

Few studies have tested baculoviruses as DNA vaccine delivery systems. Baculoviruses possess a nuclear transport signal, which can lead to efficient gene expression of inserted DNA, and the inability of baculoviruses to replicate in mammalian cells reduces pathogenicity concerns. To enhance the delivery of genes into human cells, researchers have constructed a recombinant baculovirus expressing the envelope glycoprotein of human endogenous retrovirus (HERV). The resulting AcHERV system efficiently delivers vaccine genes into human cells through type D retrovirus receptor (RDR) binding-dependent endocytosis with multiple boosting^23–27^.

In this study, we tested the ability of AcHERV to serve as a vector system for delivery of DNA encoding MERS-CoV or SARS-CoV2 antigens. To this end, we constructed various AcHERV vaccine candidates encoding full-length S, S1 subunit or RBD antigens of MERS-CoV or COVID19. Here, we report the humoral and cellular immunogenicity and prophylactic effects of AcHERV-based vaccines in challenge models of MERS-CoV and SARS-CoV2 viruses.

## Results

### Cellular expression of MERS-CoV and SARS-CoV2 antigens delivered via recombinant baculoviral vectors

**Fig. 1:**
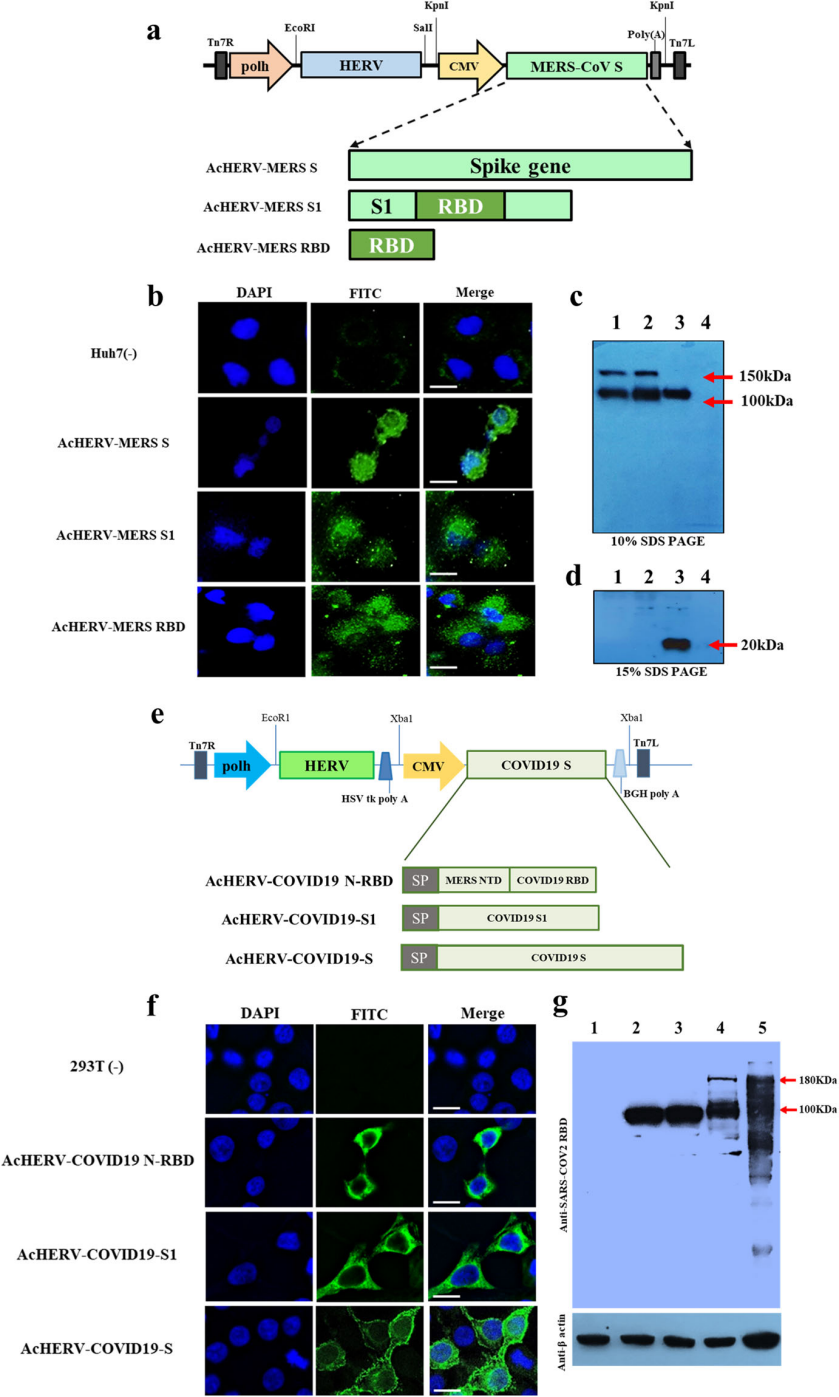
Characterization of recombinant baculoviruses encoding the MERS-CoV or SARS-CoV2 S gene. **a** Schematic diagrams of recombinant MERS baculoviruses. **b** Expression of MERS S in Huh7 cells, detected by immunofluorescence. **c** Expression of MERS S protein in 293T cells, analyzed by western blotting on 10% SDS PAGE. Lane 1: MERS pseudovirus, Lane 2: AcHERV-MERS S, Lane 3: AcHERV-MERS S1, Lane 4: uninfected 293T cells. **d** Expression of MERS S RBD protein in 293T cells, analyzed by western blotting on 15% SDS PAGE. Lane 1: AcHERV-MERS S, Lane 2: AcHERV-MERS S1, Lane 3: AcHERV-MERS RBD, Lane 4: uninfected 293T cells. **e** Schematic diagrams of recombinant COVID19 baculoviruses. **f** Expression of SARS-CoV2 S protein, detected by immunofluorescence in 293T cells. **g** Expression of COVID19 S protein in 293T cells, normalized to β-actin expression. Lane 1: uninfected cells, Lane 2: AcHERV-MERS-N-RBD, Lane 3: AcHERV-COVID19-S1, Lane 4: AcHERV-COVID19-S, Lane 5: inactivated SARS-CoV2 lysate. Scale bar = 10 μm.

A schematic diagram of recombinant baculoviruses encoding MERS-CoV antigens is shown in Fig. 1a. All transfer plasmids were constructed to express the HERV *env* under the polyhedron promoter. AcHERVs encoding MERS-CoV full-length S, S1 subunit, or RBD were constructed to express antigens under control of the CMV promoter. Immunofluorescence analyses revealed expression of MERS S protein in Huh7 cells following transduction with AcHERV-MERS-S (Fig. 1b), and western blotting showed that 293T cells transduced with AcHERV-MERS-S, AcHERV-MERS-S1 or AcHERV-MERS-RBD expressed MERS S (150 kDa), S1 (100 kDa) or RBD (20 kDa) protein, respectively (Fig. 1c).

A schematic diagram of recombinant baculoviruses encoding SARS-CoV2 is shown in Fig. 1d. AcHERV-encoding SARS-CoV2 full-length S, S1 subunit, or MERS NTD-SARS RBD (N-RBD) were constructed to express the indicated antigens under the control of the CMV promoter. Immunofluorescence assays revealed expression of SARS-CoV2 S protein in 293T cells following transduction with AcHERV-COVID19-S (Fig. 1e), and western blotting showed that 293T cells transduced with AcHERV-COVID19-S, AcHERV-COVID19-S1, or AcHERV-COVID19-N-RBD expressed SARS-CoV2 S, S1, or MERS NTD-SARS RBD protein, respectively. The molecular weights of SARS-CoV2 S, S1, and MERS N-RBD were ~180 kDa, 100 kDa, and 100 kDa, respectively (Fig. 1f).

### Immunogenicity and MERS-CoV challenge test in hDPP4 transgenic mice vaccinated with AcHERV-MERS variants

**Fig. 2:**
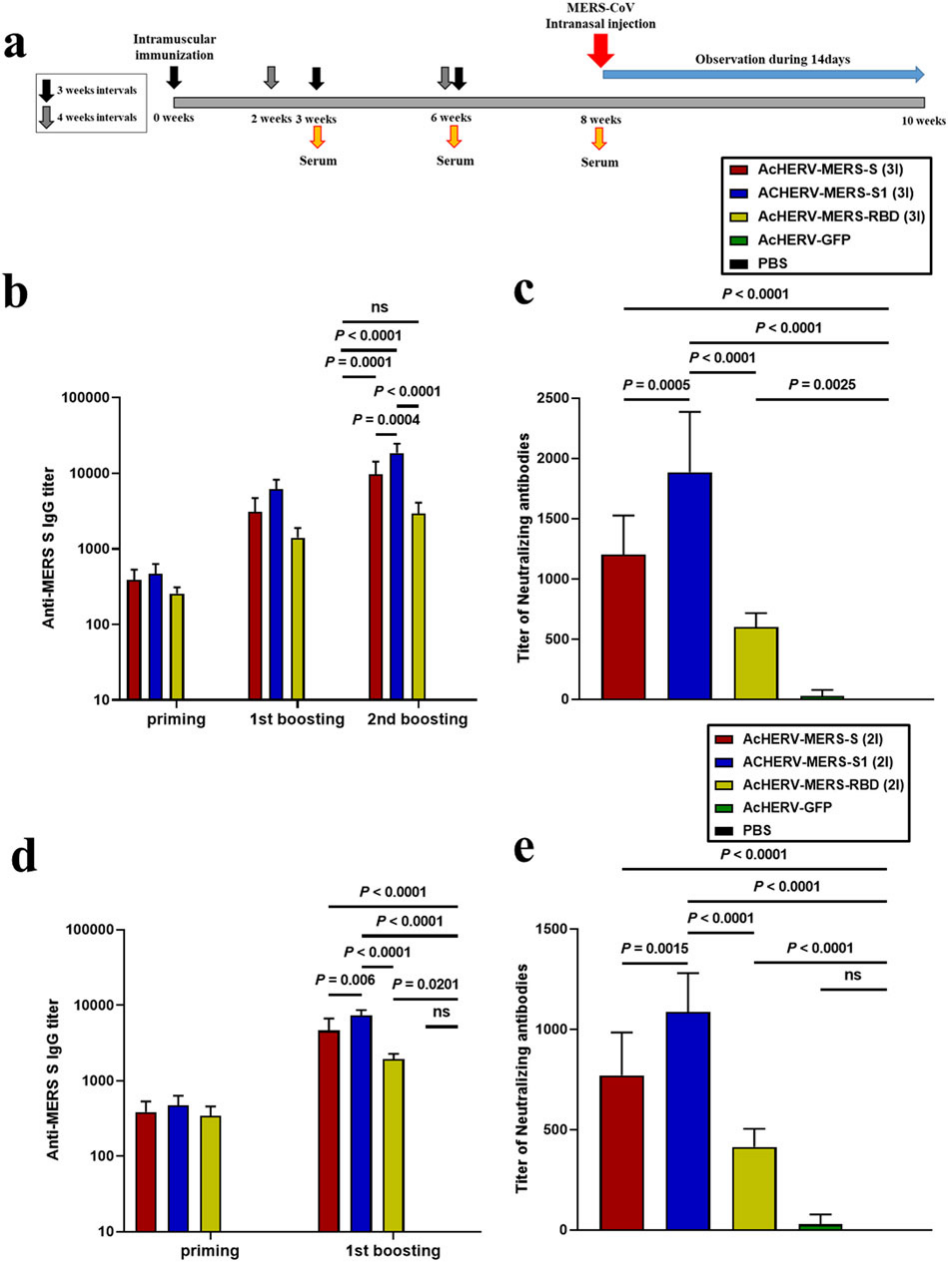
Immunogenicity of AcHERV-MERS vaccines in hDPP4 transgenic mice. **a** Vaccination schedule. hDPP4 Tg mice used for challenge experiments (Extended Table 2) were immunized three times at 3-week intervals (3×, prime-boost-boost group) or two times at a 4-week interval (2×, prime-boost group). **b** MERS-CoV-S-specific total IgG antibody responses in 3× immunization groups, determined by ELISA. **c** MERS-CoV-S-specific neutralizing antibody in 3× immunization groups. **d** MERS-CoV-S-specific total IgG antibody responses in 2× immunization groups, determined by ELISA. **e** MERS-CoV S-specific neutralizing antibody in 2× immunization groups. *P*-value determined by one-way ANOVA followed by Tukey–Kramer post hoc tests; NS, *P* > 0.05.

hDPP4 knock-in transgenic (Tg) mice develop severe lung disease in association with acute respiratory symptoms after challenge with MERS-CoV. To perform various immunization tests, we mass-produced hDPP4 Tg mice of the same age using an in vitro fertilization (IVF) approach (SI 1). Based on results of previous Ad5-hDPP4-transduced mice tests (Supplementary Table 1, Supplementary Fig. 1), we performed vaccine experiments on hDPP4 Tg mice using two different immunization schedules: three times at 3-week interval (3×) and two times at a 4-week interval (2×) (Supplementary Table 2 and Fig. 2a). Under both immunization schedules, AcHERV-MERS-S1 induced significantly higher levels of IgG and neutralizing antibodies than AcHERV-MERS-S or AcHERV-MERS-RBD (Fig. 2b–d). After the first boost, mice in the 2× group showed higher IgG titers than those in the 3× group (Fig. 2b and d). One week after the last immunization, the titers of MERS-CoV neutralizing antibodies were higher in groups treated with AcHERV-MERS-S1 compared with those in other groups (Fig. 2c, e).

**Fig. 3:**
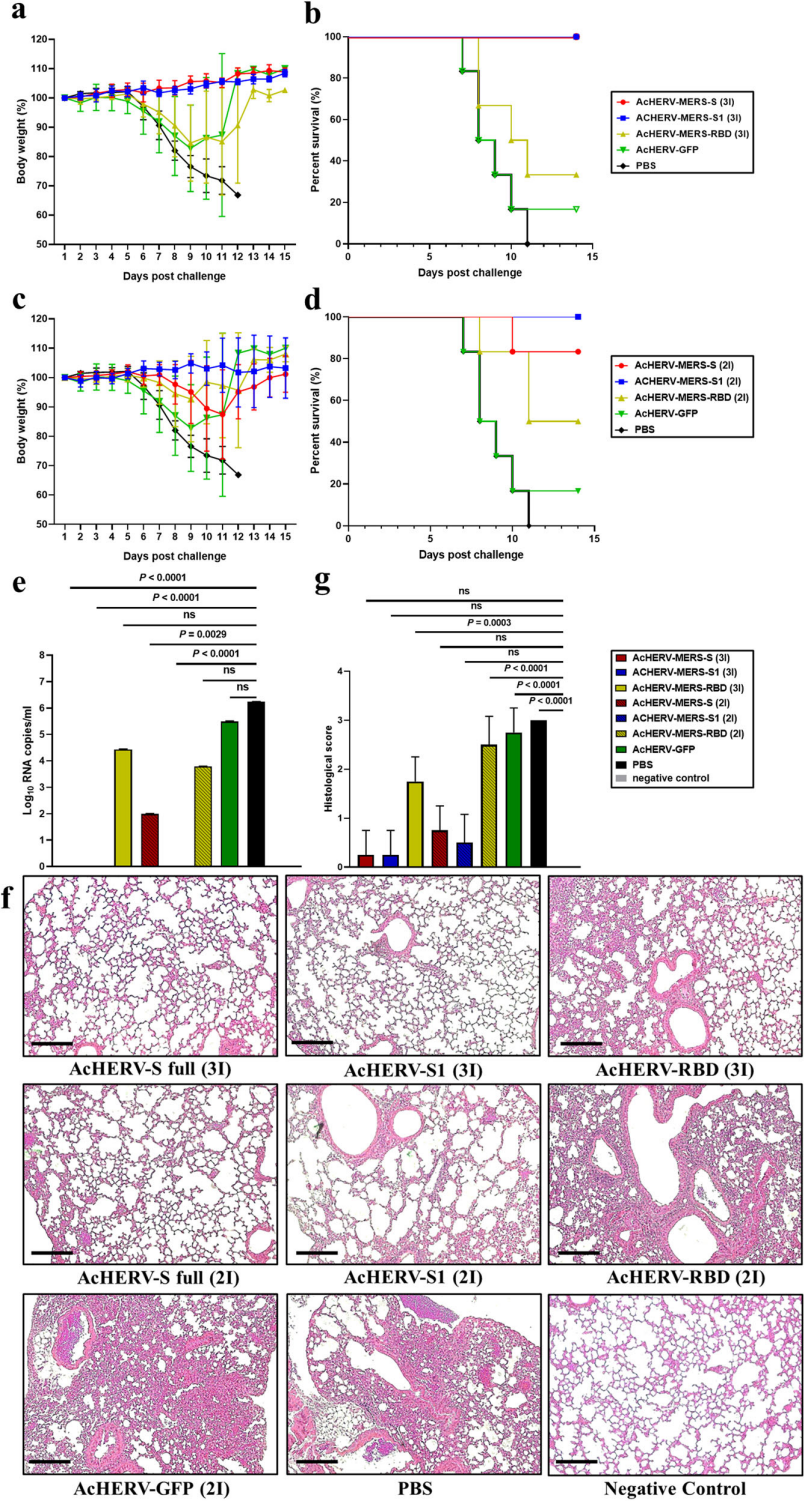
MERS-CoV challenge test in hDPP4 mice. Immunized hDPP4 mice (2× and 3× groups) were intranasally challenged with MERS-CoV and monitored for 14 days. **a** Body weight in 3× immunization groups. Results are presented as average change in body weight ± standard deviation. **b** The mouse survival rate in 3× immunization groups. **c** Body weight in 2× immunization groups. **d** The mouse survival rate in 2× immunization groups. **e** Viral lung titer in vaccinated mice (MERS full-length S, S1 subunit, and RBD), GFP, and non-vaccinated control (PBS) mice was determined by qPCR detection of the MERS-CoV N gene. **f** Assessment of H&E staining in lungs. **g** Histological evaluation of MERS-CoV vaccine groups using hDPP4 mouse. For each of group a score from 0 to 3 was given (i.e., 0 = absent, 1 = minimal, 2 = moderate or 3 = severe). Scale bar = 100 μm. *P* value determined by one-way ANOVA followed by Tukey–Kramer post hoc tests; NS, *P* > 0.05.

Changes in body weight and survival of mice treated with AcHERV-MERS vaccines were tested in hDPP4-Tg mice after challenge with MERS-CoV (Fig. 3a–d). After MERS‐CoV challenge, PBS-injected mice showed profound clinical signs on days 4–6, rapid weight loss on days 6–9, and 0% survival by day 11 (Fig. 3a, b). By day 9 post-MERS-CoV challenge, only one of six mice in the AcHERV-GFP group (16.7%) survived (Fig. 3b). In contrast, mice in the AcHERV-MERS-RBD (3×), AcHERV-MERS-RBD (2×) and AcHERV-MERS-S (2×) groups showed significant decreases in body weight, but partially recovered, exhibiting survival rates of 33.3%, 50%, and 83.3%, respectively (Fig. 3a, c). Notably, mice in AcHERV-MERS-S1 (3×), AcHERV-MERS-S1 (2×) and AcHERV-MERS-S (3×) groups showed limited body weight loss and complete protection against virus lethality (100% survival) by day 14 post challenge (Fig. 3b, d). Lungs harvested 10 days after infection showed no detectable levels of MERS-CoV in AcHERV-MERS-S1 (3×) or AcHERV-MERS S (3×) groups, determined by RT-qPCR analysis of the gene encoding the N protein. In contrast, high viral titers were observed in lungs of mice in AcHERV-MERS-RBD, AcHERV-GFP, and PBS groups (Fig. 3e). Lung tissues from mice in PBS control and AcHERV-MERS-RBD groups showed severe lesions, including the loss of pulmonary alveoli and diffuse mononuclear cell infiltration. AcHERV-MERS-S1 and AcHERV-MERS-S groups exhibited a significantly milder lung pathology following MERS-CoV infection (Fig. 3f). These results indicate that vaccination with AcHERV-MERS-S1 reduced respiratory pathologies in hDPP4-Tg mice after MERS-CoV challenge. Thus, AcHERV-MERS-S1 can be considered a vaccine candidate against MERS-CoV, which is likely to re-emerge as a pandemic in the future.

### Immunogenicity of AcHERV-COVID19 vaccines in BALB/c mice

To evaluate immune responses to AcHERV-based COVID19 vaccines, we immunized BALB/c mice intramuscularly with each recombinant baculovirus at a concentration of 2 × 10^7^ FFU/ml (Supplementary Table 3 and Supplementary Fig. 2a). Two weeks after the last vaccination, AcHERV-COVID19-S, AcHERV-COVID19-S1, and AcHERV-COVID19-N-RBD induced increases in SARS-CoV2-specific serum IgG levels in mice (Supplementary Fig. 2b). However, only AcHERV-COVID19-S elicited neutralizing antibodies specific to SARS-CoV2 (Supplementary Fig. 2c). The failure of AcHERV-COVID19-S1 to induce neutralizing antibodies contrasts with the efficacy of AcHERV-MERS-S1 and is consistent with the idea that SARS-CoV2 S1 adopts a more complex three-dimensional structure than MERS-CoV S1. To evaluate cell-mediated immune responses, we performed ELISPOT assays on splenocytes isolated 4 weeks after the last immunization. These assays revealed significantly higher levels of IFN-γ in AcHERV-COVID19-vaccinated mice compared with PBS-treated mice (*P* < 0.0001; Supplementary Fig. 2d). The levels of splenocytes-secreted IFN-γ did not significantly differ among mice treated with AcHERV-COVID19-S, AcHERV-COVID19-S1, and AcHERV-COVID19-N-RBD.

### Immunogenicity and SARS-CoV2 challenge test in Syrian golden hamsters vaccinated with AcHERV-COVID19 variants

**Fig. 4:**
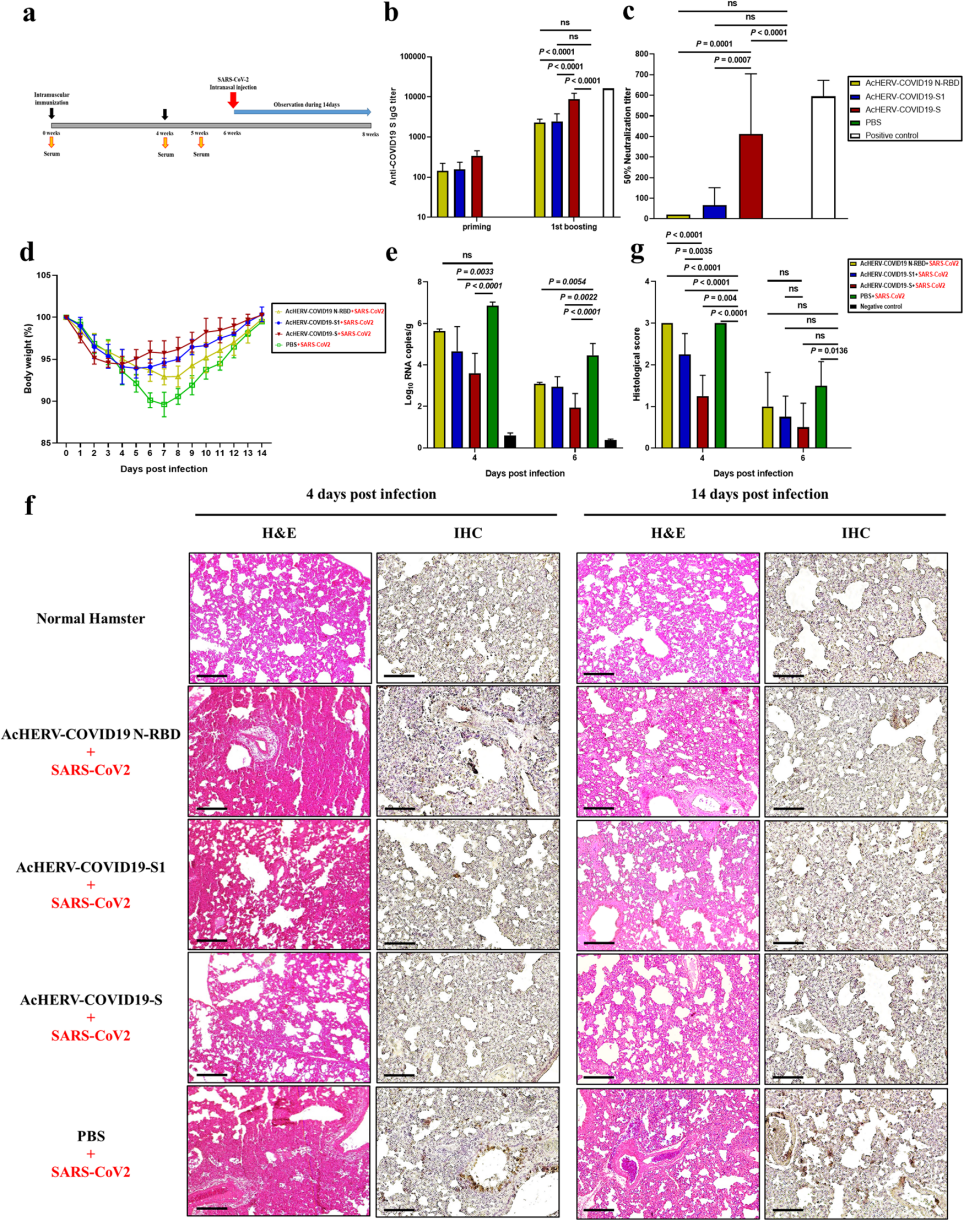
Humoral immune responses to AcHERV-COVID19 and SARS-CoV2 challenge test in hamsters. **a** Vaccination schedule. **b** SARS-CoV2-S-specific total IgG antibody responses, determined by ELISA. **c** SARS-CoV-2–specific neutralizing antibody, determined using SARS-CoV2 virus. Positive control is serum from recovered SARS-CoV2-infected patients. **d** General effects of viral infection were assessed by monitoring changes in body weight daily for 14 days. Data are presented as the average change in weight ± SD. **e** Viral lung titers of vaccinated hamsters (COVID19 full-length S, S1 subunit and MERS-N-RBD), non-vaccinated controls (PBS + SARS-CoV2 infection) and negative controls (PBS without SARS-CoV2 infection) were measured by qPCR detection of the SARS-CoV2 N gene. **f** H&E staining and immunohistochemistry. **g** Histological evaluation of SARS-CoV2 vaccine groups using hamster. For each of group a score from 0 to 3 was given (i.e., 0 = absent, 1 = minimal, 2 = moderate or 3 = severe) Scale bar = 100 μm. *P* values were determined by one-way ANOVA followed by Tukey–Kramer post hoc tests; NS, *P* > 0.05.

To test the prophylactic effect of AcHERV-COVID19 vaccines, we first used BALB/c mice. In particular, mice vaccinated with AcHERV-COVID19 showed a high cell-mediated immune response. Based on the results of BALB/c mice test (Supplementary Table 3, E Supplementary Fig. 2), we also used Syrian golden hamsters as an infectious animal model for SARS-CoV2. Hamsters were immunized twice at a 4-week interval with AcHERV-COVID19 vaccines by intramuscular injection (Supplementary Table 4) and challenged with intranasal SARS-CoV2 (Fig. 4a). AcHERV-COVID19-S induced significantly higher levels of IgG than other AcHERV-COVID19 vaccines (*P* < 0.0001; Fig. 4b). Neutralization assays revealed that vaccination with AcHERV-COVID19-S also elicited median neutralizing antibody titers that were more than 20-fold higher than those induced by vaccination with AcHERV-COVID19-S1 or AcHERV-COVID19- N-RBD (Fig. 4c).

After SARS-CoV2 challenge, the symptoms of SARS-CoV2 in hamsters were measured by decreased mobility and weight loss, but all recovered at 2 weeks without death. The hamsters of the PBS-treated group lost 10.4% of their weight by day 7 and recovered gradually. On the other hand, body weights of the AcHERV-COVID19-S group were 94.4% of their initial body weights by day 3 and recovered rapidly. In hamsters of the AcHERV-COVID19-S1 and AcHERV-COVID19-N-RBD groups, the body weight decreased 6.1% by day 5 and 7.1% by day 7, respectively (Fig. 4d). On days 4 and 6 after SARS-CoV2 challenge, viral titers were measured in lung tissue from all groups by RT-qPCR. Although AcHERV-COVID19-S did not provide complete protection, this group showed the lowest viral titer compared with other groups (Fig. 4e). Animals vaccinated with AcHERV-COVID19-S also exhibited diminished pathology compared with controls (Fig. 4f). Lung tissues in the PBS group and AcHERV-COVID19-N-RBD group showed severe lesions, including loss of pulmonary alveoli and diffuse mononuclear cell infiltration. Unlike these groups, AcHERV-COVID19-S and AcHERV-COVID19-S1 groups showed significantly milder pathologies upon SARS-CoV2 infection. These results indicate that AcHERV-COVID19-S vaccination reduced respiratory pathologies in hamsters after SARS-CoV2 challenge. When looking at the lung tissue 2 weeks after the challenge, the overall recovery was clearly in the AcHERV-COVID19 S group compared to the PBS control group.

These results indicate that the AcHERV-COVID19 S vaccine candidate can be applied to future vaccine seed viruses.

## Discussion

In this study, we demonstrated that the AcHERV system could be used to induce both humoral and cellular immunogenicity. AcHERV expressing the MERS S1 or COVID19 S gene elicited increases in antigen-specific serum IgG levels and provided 100% protection against the lethal effects of challenge. The complete protective effect of AcHERV vaccines in animal models supports the feasibility of AcHERV baculoviral vaccines against MERS-CoV and SARS-CoV2.

We found that immunization with the AcHERV-COVID19-S vaccine induced serum IgG, neutralizing antibody, and antigen-specific IFN-γ secretion. AcHERV-MERS-S1 also elicited high levels of IgG, neutralizing antibody, and T-cell immune responses (IFN-γ secretion). Unlike AcHERV-MERS-S1, AcHERV-COVID19-S1 induced relatively low neutralizing antibody titers, but still proved effective against SARC-CoV2 challenge. This implies that high cellular immunity conferred therapeutic efficacy after infection. Our findings also indicate that AcHERV-based vaccines elicited both Th1 and Th2 responses. This balanced activation of Th1 and Th2 responses by the AcHERV system would be beneficial for clearing viral loads. Recent studies have reported that the survival of patients infected with SARS-CoV2 depends on both cell-mediated immune responses (Th1) and neutralizing antibodies. Similarly, patients with strong MERS-specific CD8^+^ T-cell responses exhibit rapid virus clearance and thus a relatively brief virus exposure^28^.

Although the AcHERV system induced the expression of encoded antigens at the cellular level, the degree of induction and protection afforded by this expression differed depending on the type of encoded antigen. We found that in vivo expression of the S protein of COVID19, delivered as AcHERV-COVID19-S, was more effective than expression of the S1 protein by the AcHERV system. The converse was observed in the case of AcHERV-MERS vaccines, where greater immunogenicity was observed for the S1 protein than for the full-length S protein.

A previous study similarly reported that recombinant human Ad5 vectors encoding the shorter S1 domain of MERS-CoV induced a stronger neutralizing antibody response than those encoding full-length S. The mechanisms by which specific antigens of S or S1 proteins might induce higher immunogenicity will require further study. However, it is possible that the three-dimensional conformation of proteins expressed using the recombinant baculovirus system could affect antigen processing and interactions with MHC class proteins in antigen-presenting cells. In this context, it is possible that S1 expressed from AcHERV-COVID19-S1 might not assemble into the trimeric conformation that mimics the natural three-dimensional S protein. Current all RNA or Ad COVID19 vaccines under clinical trial are encoding full length of S^12,13,21,29^. This result underscores the importance of customized protein expression for antigenicity in vivo.

In conclusion, we have provided evidence that AcHERV can serve as a vaccine platform of MERS-CoV as well as SARS-CoV2. The AcHERV system offers several advantages as a DNA vaccine delivery vector. One major advantage is versatility. Given the propensity of infectious viruses to mutate, replacement of viral antigen S protein-encoding genes in the AcHERV genome would be a fast and effective way to adapt vaccines to emerging new pandemics in the future. Another advantage of the AcHERV system is enhanced cellular uptake of AcHERV mediated by HERV envelope proteins present on the virus surface (Supplementary Fig. 4). The other advantage of AcHERV is safety. Baculoviral genes are mostly silent in mammals^27^; the AcHERV-COVID19 S is expected to solve the safety issues of existing adenovirus vector vaccines.

## Materials and methods

### Cells

Spodoptera frugiperda 9 (Sf9) insect cells were propagated in Sf-900 II medium (Invitrogen, USA) supplemented with 3% fetal bovine serum (FBS; Gibco, USA) and 1% antibiotic-antimycotic (Invitrogen) at 28 °C. Human embryonic kidney 293 T (293T) cells, hepatocellular carcinoma (Huh7) and Africa green monkey kidney (Vero E6) cells were cultured in Dulbecco’s Modified Eagle Medium supplemented with 10% FBS (Gibco) and 1% penicillin/streptomycin (Gibco) at 37 °C.

### Virus preparation and titration

MERS-CoV (1-001-MER-IS-2015001) was provided by the Korean Centers for Disease Control and Prevention. SARS-CoV2 (BetaCoV/Korea/LCDC03/2020, NCCP No.43326) was provided by the National Culture Collection for Pathogens. All experimental procedures were performed in a Biosafety Level 3 (BL3) facility with the approval of the Konkuk University Institutional Animal Care and Use Committee. MERS-CoV was passaged in Huh7 cells and the median tissue culture infectious dose (TCID_50_/ml) was determined in the same cell line. Titration of MERS-CoV in Huh7 cells was carried out using the same protocol as used previously for influenza virus titration^30^. SAR-CoV2 was propagated and titered (TCID_50_/ml determination) in Vero E6 cells.

### Construction of AcHERV-MERS and AcHERV-COVID recombinant baculoviruses

A recombinant baculoviral vector expressing HERV *env* (pFastBac1-HERV) was previously constructed by inserting a synthetic, codon-optimized envelope gene of HERV type W (GenBank accession number NM014590; GenScript, USA) into pFastBac1 (Invitrogen). Recombinant AcHERVs encoding MERS-CoV antigens (AcHERV-MERS) were constructed by PCR-amplifying full-length S, S1 subunit, and RBD using the MERS-CoV S gene (EMC strain; provided by Prof. Shibo Jiang, Fudan University) as a template. These three genes were individually cloned into pcDNA3.1(+) containing a CMV promoter (Invitrogen), resulting in pc-MERS-S, pc-MERS-S1, and pc-MERS-RBD, respectively. Then, pFB-HERV-MERS-S, pFB-HERV-MERS-S1, and pFB-HERV-MERS-RBD were constructed by inserting pc-MERS-S, pc-MERS-S1, and pc-MERS-RBD into the pFastBac1-HERV vector. Recombinant baculoviruses were produced using the Bac-to-Bac baculovirus expression system according to the manufacturer’s instructions (Invitrogen). The scheme for constructing the recombinant baculoviruses, AcHERV-MERS-S, AcHERV-MERS-S1 and AcHERV-MERS-RBD, is shown in Fig. 1a.

Recombinant AcHERVs encoding SARS-CoV2 antigens were constructed by first synthesizing a SARS-CoV2 S gene (structurally similar to the MERS-COV S gene) that was codon-optimized for optimal expression in mammalian cells (GeneArt, USA), and then individually cloning full-length S and S1 subunit genes into pcDNA3.1 (+) plasmids containing a CMV promoter (Invitrogen). A segment of the MERS-CoV S gene corresponding to the N-terminal domain (NTD; amino acids 1–386) was fused to the SARS-CoV2 S RBD (amino acids 386–739), resulting in a MERS-CoV S NTD-SARS-CoV2 S RBD fusion (MERS-N-RBD) gene. S, S1, and MERS NTD-COVID19 RBD genes were individually cloned into the pFastBac1-HERV plasmid, and recombinant baculoviruses were produced using the Bac-to-Bac baculovirus expression system (Invitrogen) as described by the manufacturer. The scheme for constructing the recombinant baculoviruses, AcHERV-COVID19-S, AcHERV-COVID19-S1 and AcHERV-MERS-N-RBD, is shown in Fig. 1d.

These recombinant baculoviruses (AcHERVs) were further amplified by propagation in Sf9 cells. The various AcHERVs were purified by first removing debris from virus-infected cells by centrifugation at 6000 × *g* at 4 °C for 10 min. Supernatants were then overlaid on a 30% sucrose cushion and centrifuged at 40,000 rpm at 4 °C for 1.5 h in a 50.2Ti rotor (Beckman Coulter Inc., USA). The resulting pellet was resuspended in phosphate-buffered saline (PBS) and used for characterization and immunization. Baculovirus was titrated by quantitative polymerase chain reaction (qPCR) using the BacPAK qPCR Titration Kit (Takara Bio USA Inc., USA) according to the manufacturer’s instructions.

### Immunofluorescence assay and western blotting

The expression of MERS S, S1, and RBD proteins in mammalian cells was tested by infecting Huh7 cells with baculovirus at a multiplicity of infection (MOI) of 100. Three days after infection, immunofluorescence analyses and western blotting were carried out using a polyclonal antibody against MERS-CoV S protein (SICGEN, Portugal).

The expression of SARS-CoV2 S, S1, and MERS N-RBD proteins in mammalian cells was tested by infecting 293T cells with baculovirus at a MOI of 50. Three days after infection, immunofluorescence assays and western blotting were carried out using a polyclonal antibody against the spike RBD of SARS-CoV2 (Elabscience, USA).

The expression of the HERV gene in Sf9 cells following infection (3 MOI) with each baculovirus construct was assessed by western blotting using a polyclonal rabbit primary antibody specific for HERV Env (abcam, UK).

Immunofluorescence analyses were observed under a microscope (NIS-Elements-BR, Nikon or THUNDER Imager Model Organism, Leica). Western blots were developed using X-ray films. Uncropped figures are available in the Supplementary Information file. Marker bands have been indicated by their individual size. All western blots derive from the same experiment and were processed in parallel.

### Animals

BALB/c mice, human dipeptidyl peptidase 4-transgenic mice (hDPP4 Tg mice), and hamsters were used for immunogenicity testing. Six-week-old female BALB/c mice, purchased from Orient-Bio (Seungnam, Kyonggi-do, Republic of Korea), were bred under the highest filter conditions while allowing ad libitum access to water and food. Ten-week-old male Syrian golden hamsters, purchased from the Central Lab Animal (Seoul, Korea), were used as the SARS-CoV2 animal model. Human DPP4 mice were obtained from the Korea Center for Disease Control (Osong, Korea). Because the natural reproductive ability of hDPP4-Tg mice is significantly lower than that of wild-type mice, heterologous DPP4 mice for immunization and challenge tests were mass-produced through IVF. Mice were maintained and immunized in a BL2 animal facility in conformance with The Guide for the Care and Use of Laboratory Animals. Challenge experiments were performed in a BL3 animal facility at Konkuk University. All animal husbandry and experimental procedures were approved by the Konkuk University Institutional Animal Care and Use Committee (IACUC approval numbers: MERS-CoV, KU18144-1; SARS-CoV2, KU2007).

### Immunization of animal models

For immunization with AcHERV-MERS vaccines, 6-week-old female BALB/c mice and hDPP4 Tg mice were injected intramuscularly into the hind legs with 2 × 10^7^ FFU of AcHERV-MERS-S, AcHERV-MERS-S1, or AcHERV-MERS-RBD in PBS or with PBS alone (negative control). BALB/c mice were immunized three times at 3-week intervals (Supplementary Table 1), whereas hDPP4-Tg mice, used for challenge experiments (Supplementary Table 2), were immunized three times at 3-week intervals (3× group) or two times at a 4-week interval (2× group).

For immunization with AcHERV-COVID vaccines, 6-week-old female BALB/c mice and 10-week-old male Syrian golden hamsters were injected intramuscularly into the hind legs with 2 × 10^7^ FFU or 1 × 10^8^ FFU of AcHERV-COVID19-S, AcHERV-COVID19-S1, or AcHERV-MERS-N-RBD in PBS or with PBS alone (vehicle control). BALB/c mice were immunized three times at 3-week intervals (Supplementary Table 3), whereas hamsters were vaccinated two times at a 4-week interval (Supplementary Table 4). Blood samples were collected after anesthetizing mice by intramuscular injection of 40 mg/kg of Zoletil50 (Virbac Laboratories, France) and 5 mg/kg of Rompun (Bayer Korea, Republic of Korea). Blood sampled from the right external jugular vein was centrifuged at 13,000 rpm for 10 min, and the supernatant fraction was used for further assays.

### Enzyme-linked immunosorbent assay

Induction of antibodies specific for MERS S, SARS-CoV2 S, or SARS-CoV2 RBD was tested by enzyme-linked immunosorbent assay (ELISA). For detection of MERS S antibodies, a 96-well plate was coated with the MERS S protein (WooGene B&G, Korea) at 1 μg/ml by incubating for 16 h at 4 °C. After blocking with 2% bovine serum albumin in PBS for 1 h at 37 °C, the plate was washed with PBS-T, after which 1/200 serial dilutions of mouse sera (0.06 ml/well) were added and plates were incubated at room temperature for 2 h. After washing with PBS-T, plates were incubated with horseradish peroxidase (HRP)-conjugated goat anti-mouse IgG antibody (1:10000; Abcam) for 1 h at 37 °C. Next, 3,3′,5,5′-tetramethylbenzidine (TMB) substrate solution (Bio-Rad, Hercules, USA) was added and plates were incubated for 7 min, followed by addition of 1 N H_2_SO_4_ to terminate the reaction. Absorbance was recorded at 450 nm using a microplate spectrophotometer (BioTek Epoch, USA).

For detection of SARS-CoV2 S-specific antibodies, a 96-well plate was coated with the SARS-CoV2 S RBD protein (produced by our lab using an *Escherichia coli* expression system) at 1 μg/ml by incubating for 16 h at 4 °C. After blocking with 5% skim milk in PBS for 1 h at 37 °C, plates were washed with PBS-T, then 1/20 serial dilutions of mouse sera (0.06 ml/well) were added and plates were incubated at room temperature for 2 h. Wells were washed with PBS-T, then HRP-conjugated goat anti-mouse IgG secondary antibody (1:10000; Abcam, UK) was added and plates were incubated for 1 h at 37 °C. After washing, TMB solution (Bio-Rad) was added and absorbance at 450 nm was measured using a microplate spectrophotometer (BioTek Epoch).

### MERS-CoV and SARS-CoV2 neutralization assay

MERS-CoV neutralization assays were carried out using MERS-CoV pseudovirus (provided by Shibo Jang, Pudan University, China). Two weeks after the last immunization, serum samples were harvested, serially diluted twofold, and mixed 1:1 with MERS pseudovirus (3 × 10^3^ RLU [relative light units]/well). After a 1 h incubation at 37 °C, each pseudovirus–antibody mixture was added to 293T cells. After incubating at 37 °C for 3 days, cells were lysed, and luciferase content in cell lysates was determined using Beetle-Juice Luciferase Assay Firefly (PJK GmbH, Kleinblittersdorf, Germany). Neutralization titers were defined as the reciprocal of the highest serum dilution at which luciferase activity was reduced by at least 50%. Neutralization assays were performed as previously reported^31^.

SARS-CoV2 neutralization assays were performed using SARS-CoV2 in a BSL3 facility. One week after the last immunization, serum samples were harvested, serially diluted twofold, and mixed 1:1 with SARS-CoV2 virus (50 TCID_50_). After a 1-h incubation at 37 °C, each virus–antibody mixture was added to Vero E6 cells. After incubating at 37 °C for 3 days, cells were fixed and stained with Crystal violet. Serum from recovered SARS-CoV2-infected patients was used as a positive control.

### IFN-γ ELISPOT assay

The production of interferon (IFN-γ) from splenocytes of immunized mice was detected by enzyme-linked immune spot assay (ELISOPT; BD Bioscience, USA), as described by the manufacturer. Briefly, a 96-well plate was coated with 0.5 μg/ml of anti-mouse IFN-γ, and then blocked by incubating with RPMI-1640 medium containing 10% FBS and penicillin/streptomycin/l-glutamine at 37 °C. Splenocytes were seeded at 1 × 10^6^ cells per well in 100 μl of medium, and stimulated by adding MERS pseudovirus (3 × 10^3^ RLU/well) or inactivated SARS-CoV2 virus (7 × 10^5^ TCID_50_/well), constructed in our laboratory, and incubating for an additional 24 h at 37 °C. Plates were then washed with PBS-T and incubated with 0.25 μg of biotinylated anti-mouse IFN-γ detection antibodies. After 2 h, streptavidin-HRP was added to the wells, and color was developed using an AEC substrate reagent (BD Biosciences, Franklin Lakes, USA). The number of spots was counted using an ELISPOT reader (AID EliSpot Reader ver. 4; Straßberg, Germany).

### Coronavirus challenge models

#### MERS-CoV challenge in BALB/c mice transiently expressing the hDPP4 gene

Prior to challenge tests, BALB/c mice were sensitized to MERS-CoV by transiently infecting with recombinant Ad5 expressing human DPP4 (Ad5-hDPP4; provided by Professor Jae-Hwan Nam, Catholic University, Korea). Specifically, 2 weeks after the last immunization, mice were intranasally transduced with 60 μl of Ad5-hDPP4 (3 × 10^10^ IFU/ml), titrated using an Adeno-X Rapid Titer Kit (Clontech, USA). Five days later, mice were intranasally challenged with 60 μl of MERS-CoV (1 × 10^6^ TCID_50_/ml). Mouse weight was monitored daily for 8 days after challenge. At the end of this period, mice were sacrificed and lungs were harvested. Virus titer, expressed as TCID_50_/ml, was measured in Huh7 cells using the same methods as used for MERS-CoV titration.

#### MERS-CoV challenge in hDPP4-Tg mice

For MERS-CoV challenge, hDPP4-Tg mice were immunized with various recombinant vaccines or were administered vehicle control (PBS). The challenge test was conducted 10 days after the last immunization by intranasally infecting with MERS-CoV (60 μl/mouse, 1 × 10^6^ TCID_50_/ml). Body weights of mice were monitored after challenge. Mice were sacrificed 10 days post infection, and lungs were harvested for histological staining and qPCR.

#### SARS-CoV2 challenge in hamsters

Seven days after the final immunization, serum was harvested from hamsters immunized with various AcHERV-COVID vaccines. ELISAs and neutralizing assays were performed using hamster sera. Challenge tests were conducted 14 days after the last immunization by intranasally infecting with SARS-CoV2 (0.1 ml/dose, 7 × 10^6^ TCID_50_/ml). Body weights were monitored after challenge. Hamsters were sacrificed at 4, 6 and 14 days post infection, and lungs were harvested for histological staining and qPCR.

### Hematoxylin and eosin (H&E) staining

After sacrificing mice, lungs were harvested for H&E staining. Lung tissue was fixed with 10% formalin, embedded in paraffin, and sliced at a thickness of 5 μm. Lung tissue was deparaffinized according to standard procedures, stained with H&E, and examined under an optical microscope. Histological effects of the vaccine were quantitatively assessed by examining five slides per animal.

### Immunohistochemistry (IHC)

IHC was used to detect expression of SARS-CoV2 N protein. Prepared slides were deparaffinized in xylene and rehydrated through a graded ethanol series to distilled water. SARS-CoV2 N epitope retrieval was performed by heating in a pressure cooker on a steam setting for 10 min in 10 mM sodium citrate buffer, followed by incubating with 3% hydrogen peroxide for 10 min. Slides were incubated overnight at 4 °C with mouse anti-SARS-CoV N protein primary antibody (Genbody, Republic of Korea; 1:200) and then with biotinylated anti-mouse IgG (H + L) secondary antibody (BA-2000; Vector Laboratories, USA; 1:1000) for 1 h. After incubation with HRP streptavidin (SA-5004; Vector Laboratories, USA; 1:500) for 1 h, 3,3’-diaminobenzidine (DAB) substrate was added. All slides were dehydrated through a graded ethanol series to xylene and then covered with mounting solution. DAB substrate is oxidized by hydrogen peroxide in a reaction typically catalyzed by HRP. The oxidized DAB forms a brown precipitate that is then observed under an optical microscope.

### Quantitative real-time polymerase chain reaction (qRT-PCR)

MERS-CoV and SARS-CoV2 mRNA expression levels in lung tissue obtained from mice immunized with various recombinant vaccines were determined by RT-qPCR using primers for the gene encoding the N protein. RNA was extracted from each sample using the TRIzol reagent (Invitrogen), and cDNA was synthesized from total RNA using SuperScript II Reverse Transcriptase (Invitrogen). RT-qPCR was conducted using SYBRGreen, included in the BacPAK qPCR titration kit (Clontech, CA, USA), and the StepOnePlus Real-Time PCR System (Applied Biosystems, CA, USA). The RT-qPCR conditions were as follows: initial denaturation at 95 °C for 30 s, followed by 40 cycles of 3 s at 95 °C and 25 s at 60 °C, and 1 cycle of 15 s at 95 °C, 60 s at 60 °C, and 15 s at 95 °C. The single amplified product was confirmed using agarose gel electrophoresis. The total mRNA copy number was quantified by reference to a standard curve prepared using serial dilutions of the pGEM-T vector containing a partial N gene of MERS-CoV or SARS-CoV2.

### Genomic DNA isolation and PCR methods

Five weeks after birth, hDPP4 mice were individually selected, their genomic DNA was extracted (Supplementary Fig. 3). Genomic DNA was isolated from mouse-tail tissue using the commercial kit DNeasy^®^ Blood & Tissue (Qiagen) following the protocol provided by the manufacturer. The hDPP4 gene was identified by PCR using a specific primer set (forward primer: 5′CGC TAT TAC CAT GGT GAT GCG 3′, reverse primer: 5′AGC TGT AGC ATC ATC TGT GCC 3′), obtaining an amplicon size of 984 bps. The following PCR conditions were used: 94 °C for 5 min followed by 35 cycles at 94 °C for 1 min, 55 °C for 1 min, 72 °C for 1 min, and final extension at 72 °C for 10 min. The single amplified product was confirmed using agarose gel electrophoresis.

### GFP fluorescence and FACS analysis

To evaluate the AcHERV delivery system, 293T cells were infected with AcHERV-cmvGFP or Ac-cmvGFP (MOI 50). Mammalian cells were transduced with recombinant baculoviruses and analyzed by measuring GFP fluorescence by FACS analysis at 48 h post transduction (Supplementary Fig. 4). GFP fluorescence analyses were observed under a microscope (NIS-Elements-BR, Nikon). FACS analyses were observed FACSCalibur (Becton Dickinson, USA).

### Statistical analysis

All statistical analyses were performed using GraphPad Prism 8.0.2 (GraphPad Software), and data are presented as means ± standard deviation (SD). Comparisons of data between groups were performed using one-way analysis of variance (ANOVA) followed by Tukey–Kramer post hoc tests. *P* values <0.05 were considered statistically significant.

### Reporting summary

Further information on research design is available in the Nature Research Reporting Summary linked to this article.

## Supplementary information

Supplementary Information

Reporting Summary

## Data Availability

The data that support the findings of this study are available from the authors on reasonable request, see author contributions for specific data sets.
