# Supplementary material for: Human endogenous retrovirus-enveloped baculoviral DNA vaccines against MERS-CoV and SARS-CoV2
Source: NPJ Vaccines. 2021 Mar 19;6:37. doi: 10.1038/s41541-021-00303-w (PMC7979866; doi:10.1038/s41541-021-00303-w)
Supplement: Supplementary file 1 — Supplementary Information [file 41541_2021_303_MOESM1_ESM.pdf]

Supplementary Figure 1.

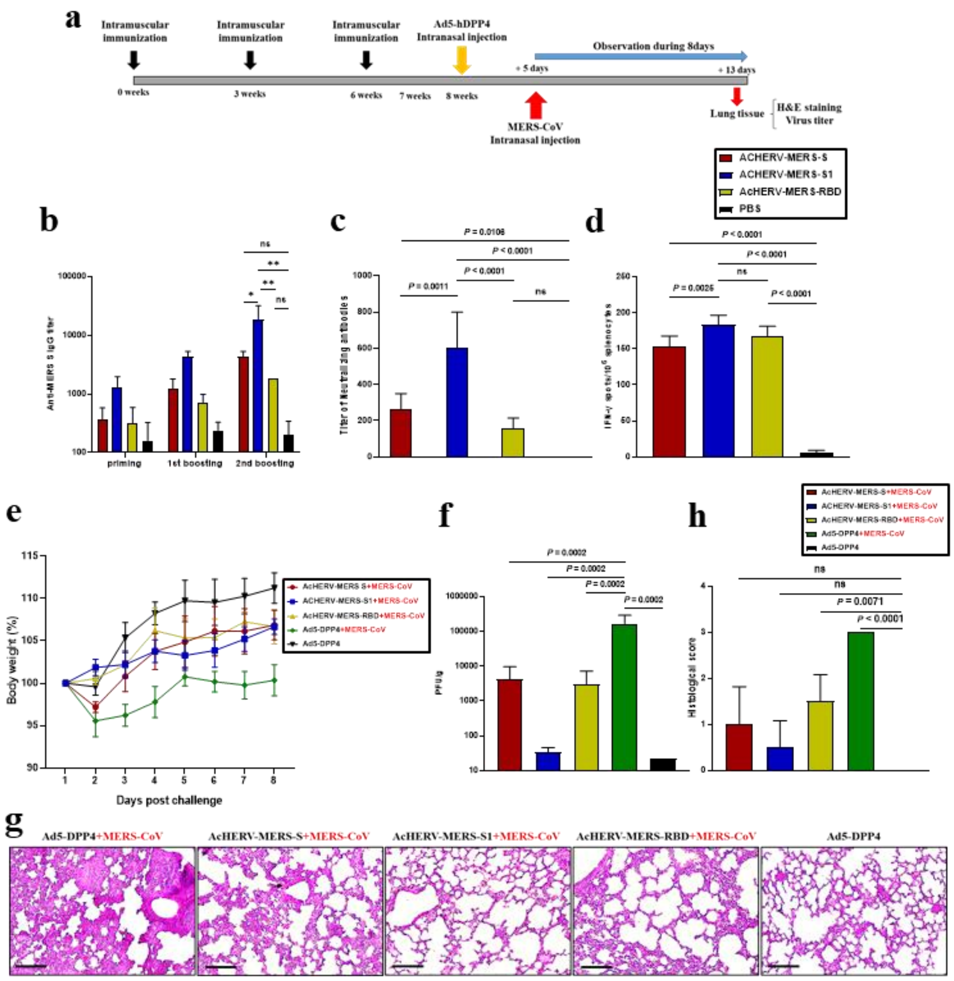

Supplementary Figure 1. Immunogenicity and MERS-CoV challenge test in vaccinated, Ad5-hDPP4–transduced BALB/c mice. a, Vaccination schedule. b, IgG antibody responses in sera from vaccinated mice, determined by ELISA. IgG endpoint titers are presented as means ± standard deviation (SD) of 7 mice per group (***P* = 0.0032). c, MERS-CoV-S–specific neutralizing antibody in mice immunized with recombinant baculovirus and in vehicle control (PBS) mice. d, Induction of MERS-CoV pseudovirus-specific T cells using an ELISPOT assay. e, Protection against MERS-CoV challenge after immunization. Results are presented as average change in body weight ± standard deviation. f, Viral lung titer in vaccinated mice and non-vaccinated controls (Ad5-DPP4). g, Assessment of H&E staining in lungs. h. Histological evaluation of MERS-CoV vaccine groups using Ad5-hDPP4 transduced BALB/c mice. For each of group a score from 0 to 3 was given (i.e., 0 = absent, 1 = minimal, 2 = moderate or 3 = severe). Scale bar = 100 μm. *P*-value determined by one-way ANOVA followed by Tukey-Kramer post hoc tests; NS, *P* > 0.05.

Supplementary Figure 2.

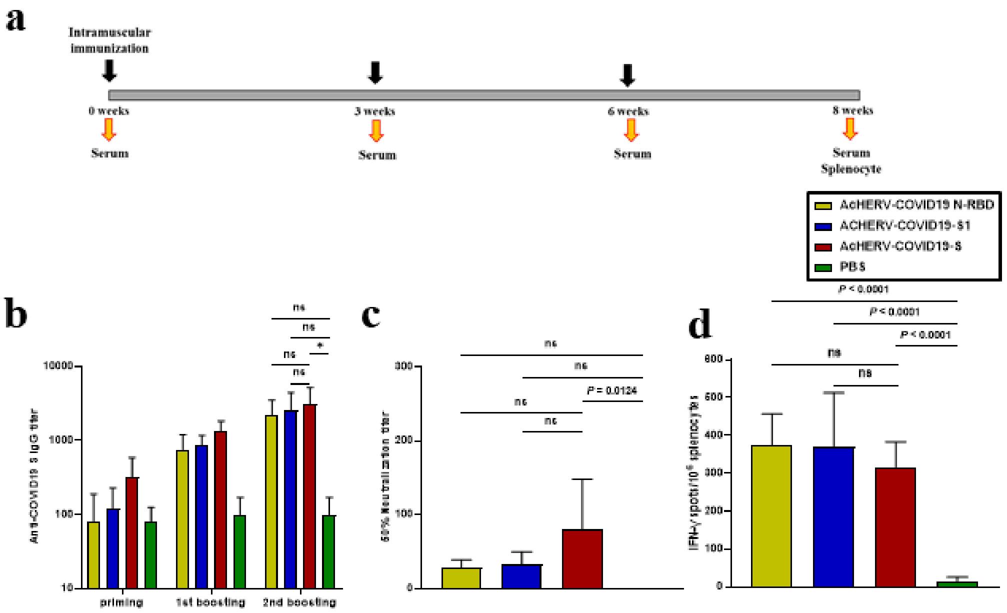

Supplementary Figure 2. Immunogenicity of AcHERV-COVID19 vaccines in BALB/c mice. a, Vaccination schedule. b, IgG antibody responses in sera from vaccinated mice, determined by ELISA. IgG endpoint titers are presented as means ± standard deviation (SD) of seven mice per group (**P* = 0.0346). c, COVID-19 neutralization assay. d, Induction of SARS-CoV2–specific T cells using an ELISPOT assay. *P*-values were determined by one-way ANOVA followed by Tukey-Kramer post hoc tests; NS, *P* > 0.05.

Supplementary Figure 3.

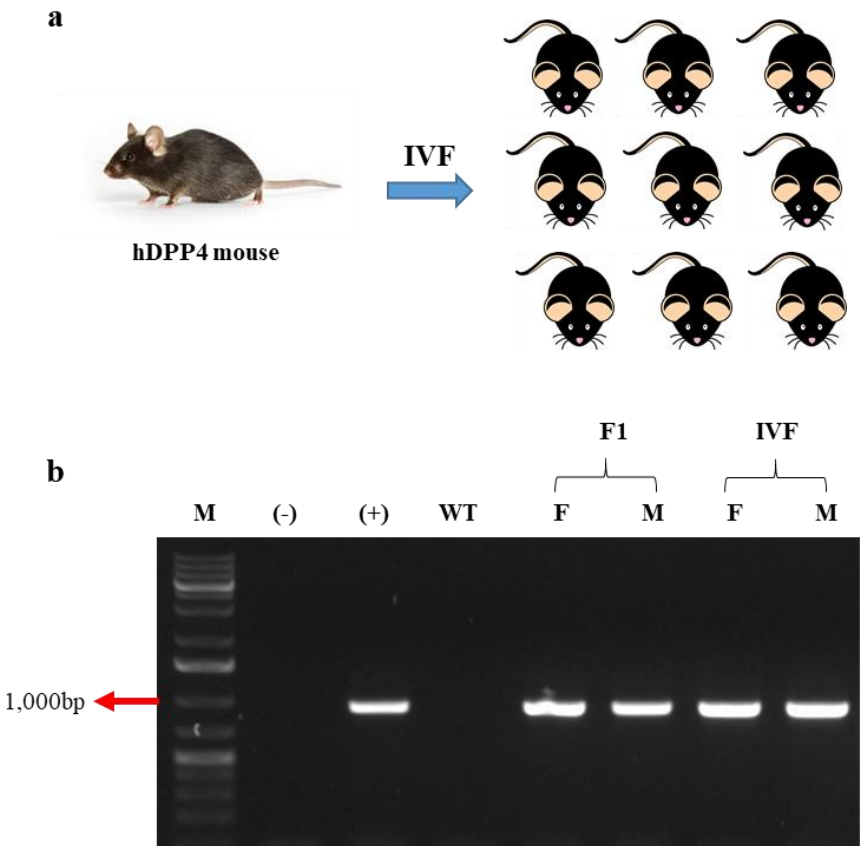

Supplementary Figure 3. Mass production of hDPP4 mice using IVF methods. **a,** hDPP4 mice used for immunization with the MERS-CoV vaccine were obtained by IVF. b, Five weeks after birth, mice were individually selected, their genomic DNA was extracted. The hDPP4 gene was identified by PCR using a specific primer set (forward primer: 5'CGC TAT TAC CAT GGT GAT GCG 3', reverse primer: 5'AGC TGT AGC ATC ATC TGT GCC 3'), obtaining an amplicon size of 984 bps. The following PCR conditions were used: 94˚C for 5 min followed by 35 cycles at 94˚C for 1 min, 55˚C for 1 min, 72˚C for 1 min, and final extension at 72˚C for 10 min. M: DNA Ladder, (-): PCR negative Control, (+): positive control (provided hDPP4 genomic DNA), WT: C57Bl/6, F: female hDPP4 mouse produced with F1 or IVF, M: male hDPP4 mouse produced with F1 or IVF. F1 is first generation. IVF is in vitro fertilization.

Supplementary Figure 4.

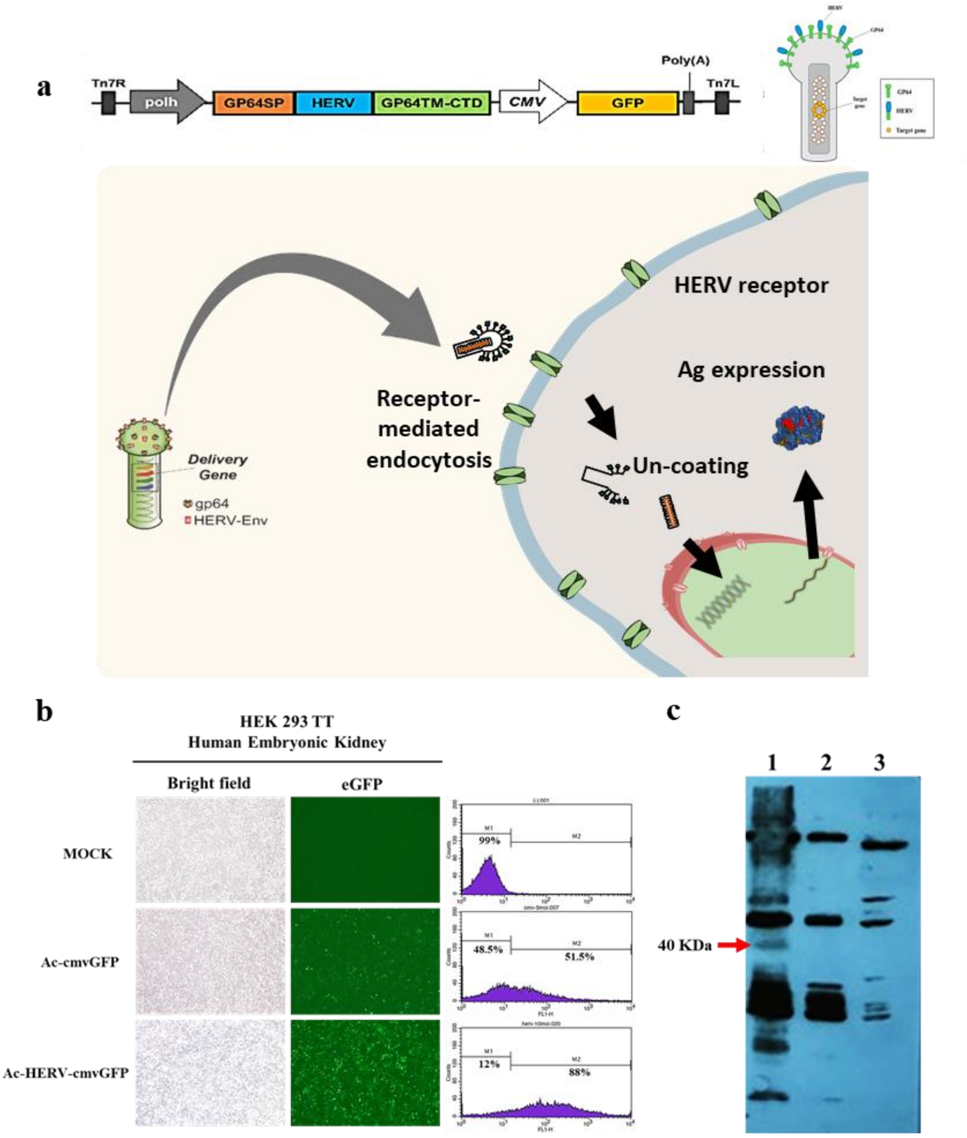

Supplementary Figure 4. Evaluation of the AcHERV DNA vaccine system. a, Schematic diagram of the transfer plasmid (derived from pFastBac1) used for construction of the AcHERV system. The polyhedrin gene promoter was used to drive expression of the HERV gene in all plasmids, and the CMV promoter was used to drive expression of the GFP gene. A poly (A) tail was inserted at the end of each target gene. b, Expression of GFP in 293TT cells. 293TT cells were infected with AcHERV-cmvGFP or Ac-cmvGFP (MOI 50). Mammalian cells were transduced with recombinant baculoviruses and analyzed by measuring GFP fluorescence by FACS analysis at 48 hours post transduction. c, Expression of HERV in Sf9 insect cells. Expression of the HERV gene in Sf9 cells following infection with each baculovirus construct was assessed by Western blotting using a polyclonal rabbit primary antibody specific for HERV Env. The molecular weight of HERV is approximately 40 kDa. Lane 1: AcHERV-cmvGFP, Lane 2: Ac-cmvGFP; Lane 3: Sf9 cells.

Supplementary Figure 5.

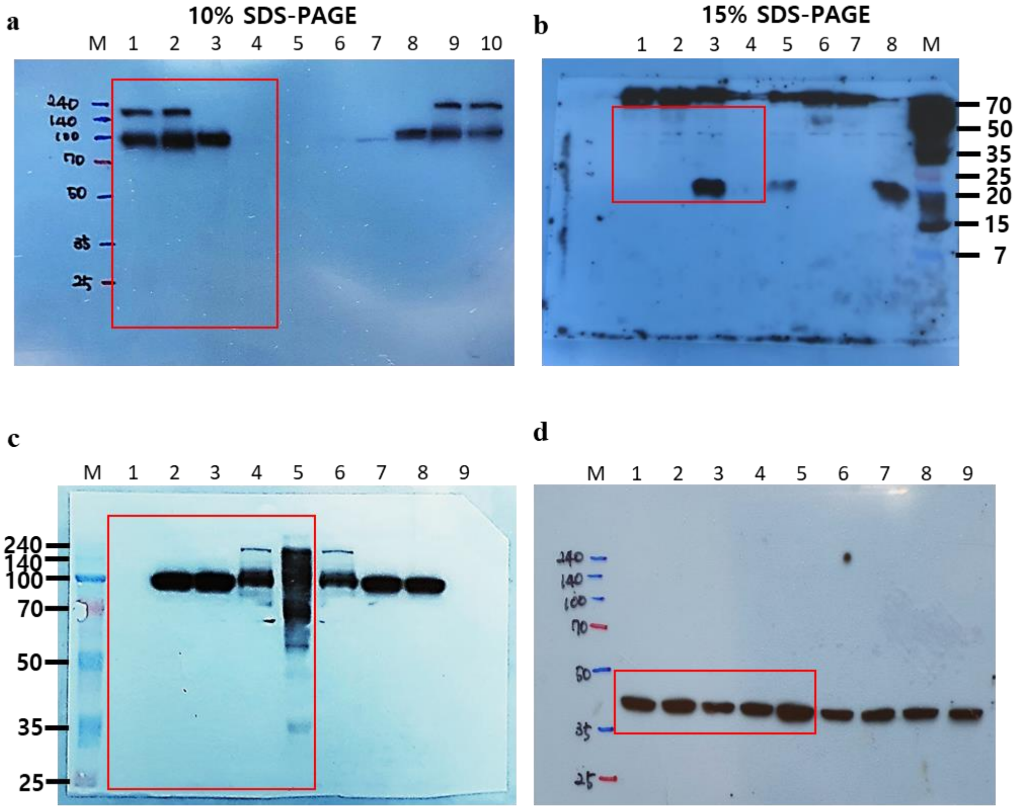

Supplementary Figure 5. Full image of western blots. a, Figure 2c (10% SDS-PAGE). Lane 1: MERS pseudovirus, Lane 2: AcHERV-MERS S, Lane 3: AcHERV-MERS S1, Lane 4: uninfected 293T cells, Lane 5: blank, Lane 6: blank, Lane 7: uninfected 293T cells, Lane 8: AcHERV-MERS S1, Lane 9: AcHERV-MERS S, Lane 10: MERS pseudovirus. b, Figure 2d (15% SDS-PAGE). Lane 1: AcHERV-MERS S, Lane 2: AcHERV-MERS S1, Lane 3: AcHERV MERS RBD, Lane 4: uninfected 293T cells, Land 5: AcHERV MERS RBD, Lane 6: AcHERV-MERS S1, Lane 7: AcHERV-MERS S, Lane 8: uninfected 293T cells, M is protein marker. c, Figure 2g (SARS-CoV2 S protein). Lane 1: uninfected cells, Lane 2: AcHERV-MERS-N-RBD, Lane 3: AcHERV-COVID19-S1, Lane 4: AcHERV-COVID19-S, Lane 5: inactivated SARS-CoV2 lysate, Lane 6: AcHERV-COVID19-S, Lane 7: AcHERV-COVID19-S1, Lane 8: AcHERV-MERS-N-RBD, Lane 9: uninfected cells. d, Figure 2g (β-actin). Lane 1: uninfected cells, Lane 2: AcHERV-MERS-N-RBD, Lane 3: AcHERV-COVID19-S1, Lane 4: AcHERV-COVID19-S, Lane 5: inactivated SARS-CoV2 lysate, Lane 6: AcHERV-COVID19-S, Lane 7: AcHERV-COVID19-S1, Lane 8: AcHERV-MERS-N-RBD, Lane 9: uninfected cells. Red box is indicated each of figure image.

Supplementary Figure 6.

a

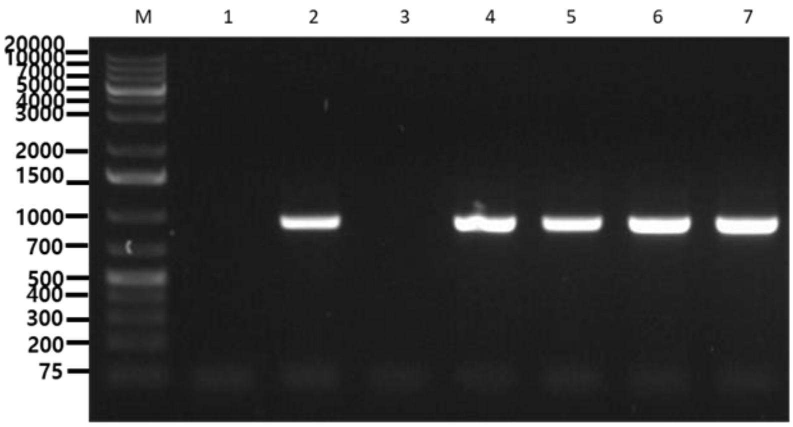

b

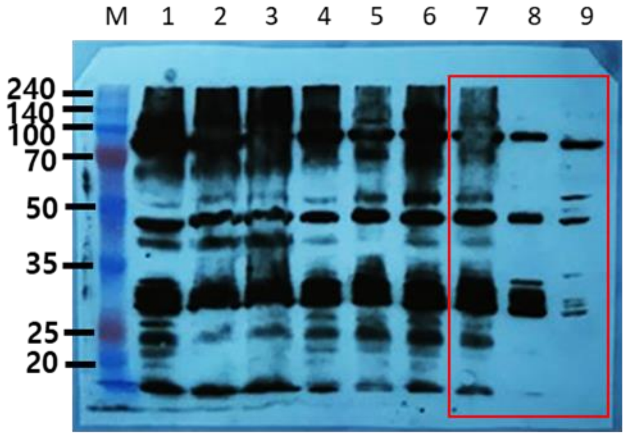

Supplementary Figure 6. Full image of agarose gel and western blots. a. Supplementary Information 1b. M: DNA Ladder, Lane 1: PCR negative Control, Lane 2: positive control (provided hDPP4 genomic DNA), Lane 3: wild type C57Bl/6, Lane 4: female hDPP4 mouse produced with F1, Lane 5: male hDPP4 mouse produced with F1. Lane 6: female hDPP4 mouse produced with IVF. Lane 7: male hDPP4 mouse produced with F1 or IVF. b, Supplementary Information 2c. Lane 1: AcHERV-COVID19-S #1, Lane 2: AcHERV-COVID19-S #2, Lane 3: AcHERV-COVID19-S1 #1, Lane 4: AcHERV-COVID19-S1 #2, Lane 5: AcHERV-MERS-N-RBD #1, Lane 6: AcHERV-MERS-N-RBD #2, Lane 7: AcHERV-cmvGFP, Lane 8: Ac-cmvGFP, Lane 9: sf9 cells. Red box is indicated each of figure image.

Supplementary Table 1.

Groups	Immunization (3 times, 3-week intervals)			
	**Construct (2×10^7 FFU/ml)**	**Ad5-hDPP4 (3×10^{10} FFU/ml)**	**MERS-CoV (1×10^6 $TCID_{50}$/ml)**	**No. of mice**
**Group 1**	**AcHERV-MERS-S**	**O**	**O**	**7**
**Group 2**	**AcHERV-MERS-S1**	**O**	**O**	**7**
**Group 3**	**AcHERV-MERS-RBD**	**O**	**O**	**7**
**Group 4**	**PBS**	**O**	**O**	**7**
**Group 5**	**PBS**	**O**	**X**	**7**

Supplementary Table 1. AcHERV-MERS immunization and challenge test in mice transduced with Ad5-hDPP4. Two weeks following the last immunization, mice vaccinated with recombinant baculoviruses or administered PBS (control) were intranasally transduced with 60 µl of Ad5-hDPP4 (3×10^{10} IFU/ml). After 5 days, Ad5-hDPP4–transduced mice were intranasally challenged with 60 µl of MERS-CoV (1×10^6 $TCID_{50}$/ml). Mouse body weights were monitored daily for 8 days after challenge.

Supplementary Table 2.

Groups	Immunization				
	Construct (2×10^7 FFU/ml)	Schedule	MERS-CoV (1×10^6 $TCID_{50}$/ml)	No. of mice	Survival (%)
Group 1	AcHERV-MERS S(3X)	3 weeks, 3 times	O	6	6/6 (100%)
Group 2	AcHERV-MERS S1(3X)	3 weeks, 3 times	O	6	6/6 (100%)
Group 3	AcHERV-MERS RBD(3X)	3 weeks, 3 times	O	6	2/6 (33.3%)
Group 4	AcHERV-MERS S(2X)	4 weeks, 2 times	O	6	5/6 (83.3%)
Group 5	AcHERV-MERS S1(2X)	4 weeks, 2 times	O	6	6/6 (100%)
Group 6	AcHERV-MERS RBD(2X)	4 weeks, 2 times	O	6	3/6 (50%)
Group 7	AcHERV-GFP	4 weeks, 2 times	O	6	1/6 (16.7%)
Group 8	PBS	-	O	6	0/6 (0%)

Supplementary Table 2. AcHERV-MERS immunization and challenge test in hDPP4 transgenic mice. Fourteen days after the last immunization, hDPP4 Tg mice were challenged with MERS-CoV via the intranasal route (60 μl/mice, 1×10^6 $TCID_{50}$/ml). Mouse body weight was monitored daily for 14 days after challenge.

Supplementary Table 3.

Groups	Immunization		
	Construct (2×10^7 FFU/ml)	Schedule	No. of mice
Group 1	AcHERV-COVID19-S	3 weeks, 3 times	5
Group 2	AcHERV-COVID19-S1	3 weeks, 3 times	5
Group 3	AcHERV-COVID19-N-RBD	3 weeks, 3 times	5
Group 4	PBS	-	5

Supplementary Table 3. AcHERV-COVID immunization schedule in BALB/c mice

Supplementary Table 4.

Groups	Immunization			
	Construct (1×10^8 FFU/ml)	Schedule	SARS-CoV2 (7×10^6 $TCID_{50}$/ml)	No. of hamsters
Group 1	AcHERV-COVID19-S	4 weeks, 2 times	O	7
Group 2	AcHERV-COVID19-S1	4 weeks, 2 times	O	7
Group 3	AcHERV-COVID19-N-RBD	4 weeks, 2 times	O	7
Group 4	PBS	-	O	7

Supplementary Table 4. AcHERV-COVID immunization and challenge test in Syrian golden hamsters. Fourteen days after the last immunization, hamsters were challenged with SARS-COV2 via the intranasal route (100 µl/mouse, 7×10^6 $TCID_{50}$/ml). Hamster body weight was monitored daily for 14 days after challenge.
