# Supplementary material for: Human endogenous retrovirus-enveloped baculoviral DNA vaccines against MERS-CoV and SARS-CoV2
Source: NPJ Vaccines. 2021 Mar 19;6:37. doi: 10.1038/s41541-021-00303-w (PMC7979866; doi:10.1038/s41541-021-00303-w)
Supplement: Supplementary file 2 — Reporting Summary [file 41541_2021_303_MOESM2_ESM.pdf]

nature research

Corresponding author(s): Young Bong Kim

Last updated by author(s): Feb 18, 2021

Reporting Summary

Nature Research wishes to improve the reproducibility of the work that we publish. This form provides structure for consistency and transparency in reporting. For further information on Nature Research policies, see our Editorial Policies and the Editorial Policy Checklist.

Statistics

For all statistical analyses, confirm that the following items are present in the figure legend, table legend, main text, or Methods section.

n/a	Confirmed	
☐	☒	The exact sample size (*n*) for each experimental group/condition, given as a discrete number and unit of measurement
☐	☒	A statement on whether measurements were taken from distinct samples or whether the same sample was measured repeatedly
☐	☒	The statistical test(s) used AND whether they are one- or two-sided<br>*Only common tests should be described solely by name; describe more complex techniques in the Methods section.*
☒	☐	A description of all covariates tested
☒	☐	A description of any assumptions or corrections, such as tests of normality and adjustment for multiple comparisons
☐	☒	A full description of the statistical parameters including central tendency (e.g. means) or other basic estimates (e.g. regression coefficient) AND variation (e.g. standard deviation) or associated estimates of uncertainty (e.g. confidence intervals)
☐	☒	For null hypothesis testing, the test statistic (e.g. *F*, *t*, *r*) with confidence intervals, effect sizes, degrees of freedom and *P* value noted<br>*Give P values as exact values whenever suitable.*
☒	☐	For Bayesian analysis, information on the choice of priors and Markov chain Monte Carlo settings
☒	☐	For hierarchical and complex designs, identification of the appropriate level for tests and full reporting of outcomes
☒	☐	Estimates of effect sizes (e.g. Cohen's *d*, Pearson's *r*), indicating how they were calculated

Our web collection on statistics for biologists contains articles on many of the points above.

Software and code

Policy information about availability of computer code

Data collection	Nikon Elements (NIS ElementsAR ver. 4.6.0.) was used to acquire images. GraphPad Prism 8.0.2 (GraphPad Software) was used to statistical analysis.
Data analysis	The image picture was captured using software (NIS ElementsAR ver. 4.6.0.).<br>All data were graphed and statistically analyzed using GraphPad Software.

For manuscripts utilizing custom algorithms or software that are central to the research but not yet described in published literature, software must be made available to editors and reviewers. We strongly encourage code deposition in a community repository (e.g. GitHub). See the Nature Research guidelines for submitting code & software for further information.

Data

Policy information about availability of data

All manuscripts must include a data availability statement. This statement should provide the following information, where applicable:

- Accession codes, unique identifiers, or web links for publicly available datasets
- A list of figures that have associated raw data
- A description of any restrictions on data availability

The data that support the findings of this study are available from the authors on reasonable request, see author contributions for specific data sets.

Field-specific reporting

Please select the one below that is the best fit for your research. If you are not sure, read the appropriate sections before making your selection.

☒ Life sciences ☐ Behavioural & social sciences ☐ Ecological, evolutionary & environmental sciences

For a reference copy of the document with all sections, see nature.com/documents/nr-reporting-summary-flat.pdf

Life sciences study design

All studies must disclose on these points even when the disclosure is negative.

Sample size	For the evaluation of the immunogenicity of the vaccine, a minimum number of animals was chosen of five. In the case of the virus challenge vaccination experiment, the number of animals was determined to be 6 or more. We determined that the vaccine efficacy evaluation was sufficient due to comparison with the negative control.
Data exclusions	Data were not excluded from analysis.
Replication	All replication attempts were successful and results were consistently drawn through repeated experiments.
Randomization	All experimental animals were distinguished through object recognition marks such as individual object numbering by groups. There was no requirement for randomization.
Blinding	Blinding was not possible as experimental conditions. Each experimental data has specificity. For example, ELSIA and nuetrlizing assays confirm a specific antibody response, and in the case of viral challenge, also observe protective performance.

Reporting for specific materials, systems and methods

We require information from authors about some types of materials, experimental systems and methods used in many studies. Here, indicate whether each material, system or method listed is relevant to your study. If you are not sure if a list item applies to your research, read the appropriate section before selecting a response.

Materials & experimental systems

n/a	Involved in the study	
☐	☒	Antibodies
☐	☒	Eukaryotic cell lines
☒	☐	Palaeontology and archaeology
☐	☒	Animals and other organisms
☒	☐	Human research participants
☒	☐	Clinical data
☒	☐	Dual use research of concern

Methods

n/a	Involved in the study	
☒	☐	ChIP-seq
☒	☐	Flow cytometry
☒	☐	MRI-based neuroimaging

Antibodies

Antibodies used	Anti-MERS-CoV S (SICGEN, AB0161-200), used at 1:1000<br>Anti-SARS-CoV2 S RBD (Elabscience, E-AB-V1006), used at 1:1000<br>Anti-HERV (abcam, ab45500), used at 1:1000<br>Rabbit anti-Goat (abcam, ab6741), used at 1:2000 from 2mg/mL stock solution<br>Goat anti-Rabbit (abcam, ab6721), used at 1:2000 from 2mg/mL stock solution
Validation	Anti-MERS-CoV (SICGEN, AB0161-200) antibody has been developed in SICGEN for demonstrating immunoblotting.<br>Anti-SARS-CoV2 S RBD (Elabscience, E-AB-V1006) antibody has been developed in Elabscience for demonstrating immunoblotting.<br>Anti-HERV (abcam, ab45500) antibody has been developed in abcam for demonstrating immunoblotting.

Eukaryotic cell lines

Policy information about cell lines

Cell line source(s)	Sf9 cells (Insect)<br>293T cells (Human)<br>Huh7 cells (Human)<br>Vero E6 cells (Monkey)
Authentication	None of the cell lines have been authenticated.

Mycoplasma contamination	Cell lines were tested for mycoplasma contamination using TaKaRa PCR Mycoplasma Detection Set (Takara, cat# 6601) and no indication of contamination was observed.
Commonly misidentified lines (See ICLAC register)	No commonly misidentified cell lines were used.

Animals and other organisms

Policy information about studies involving animals; ARRIVE guidelines recommended for reporting animal research

Laboratory animals	Wild-type BALB/c mice (female) age 6W were used for vaccine evaluation.<br>Human DPP4 transgenic mice (female) age 6W were used for MERS-CoV infection model.<br>Syrian golden hamter (male) age 10W were used for SARS-CoV2 infection model.
Wild animals	No wild animals were used in this study.
Field-collected samples	No field-collected samples were used in this study.
Ethics oversight	All animal husbandry and experimental procedures were approved by the Konkuk University Institutional Animal Care and Use Committee (IACUC approval numbers: MERS-CoV, KU18144-1; SARS-CoV2, KU2007)

Note that full information on the approval of the study protocol must also be provided in the manuscript.
